# Phloem Metabolites of *Prunus* Sp. Rather than Infection with *Candidatus* Phytoplasma Prunorum Influence Feeding Behavior of *Cacopsylla pruni* Nymphs

**DOI:** 10.1007/s10886-020-01148-8

**Published:** 2020-01-22

**Authors:** Jannicke Gallinger, Jürgen Gross

**Affiliations:** 1 Laboratory of Applied Chemical Ecology, Institute for Plant Protection in Fruit Crops and Viticulture, Federal Research Centre for Cultivated Plants, Julius Kühn-Institut, Dossenheim, Germany; 2 Plant Chemical Ecology, Technical University of Darmstadt, Schnittspahnstr. 4, 64287 Darmstadt, Germany

**Keywords:** Plant-insect interaction, European stone fruit yellows, Vector development, Phytobiome, Phloem composition, Electropenetrography, Phytoplasma

## Abstract

Phytoplasmas are specialized small bacteria restricted to the phloem tissue and spread by hemipterans feeding on plant sieve tube elements. As for many other plant pathogens, it is known that phytoplasmas alter the chemistry of their hosts. Most research on phytoplasma-plant interactions focused on the induction of plant volatiles and phytohormones. Little is known about the influence of phytoplasma infections on the nutritional composition of phloem and consequences on vector behavior and development. The plum psyllid *Cacopsylla pruni* transmits ‘*Candidatus* Phytoplasma prunorum’, the causing agent of European Stone Fruit Yellows (ESFY). While several *Prunus* species are susceptible for psyllid feeding, they show different responses to the pathogen. We studied the possible modulation of plant-insect interactions by bacteria-induced changes in phloem sap chemistry. Therefore, we sampled phloem sap from phytoplasma-infected and non-infected *Prunus persica* and *Prunus insititia* plants, which differ in their susceptibility to ESFY and psyllid feeding. Furthermore, the feeding behavior and development of *C. pruni* nymphs was compared on infected and non-infected *P. persica* and *P. insititia* plants. Phytoplasma infection did not affect phloem consumption by *C. pruni* nymphs nor their development time. In contrast, the study revealed significant differences between *P. insititia* and *P. persica* in terms of both phloem chemistry and feeding behavior of *C. pruni* nymphs*.* Phloem feeding phases were four times longer on *P. insititia* than on *P. persica*, resulting in a decreased development time and higher mortality of vector insects on *P. persica* plants. These findings explain the low infestation rates of peach cultivars with plum psyllids commonly found in field surveys.

**Electronic supplementary material:**

The online version of this article (10.1007/s10886-020-01148-8) contains supplementary material, which is available to authorized users.

## Introduction

Phytoplasmas are phloem-restricted plant pathogenic bacteria, causing severe diseases in different plant species. Many of these phytoplasma-induced diseases affect agricultural crops (Bertaccini et al. 2014), resulting in high economic losses in crop production all over the world (Smith 1997). For example, the causal agent of the European stone fruit yellows (ESFY), *‘Candidatus* Phytoplasma prunorum’, infects different species of the genus *Prunus*. Infected trees suffer from severe symptoms, yield poorly, and exhibit dieback and decline (Kison and Seemüller 2001; Marcone et al. 1996; Nečas et al. 2017). Several *Prunus* species are susceptible to ‘*Ca*. P. prunorum’ but vary in degree of symptom expression (Carraro et al. 2004a; Jarausch et al. 2000). Peaches, apricots and Japanese plums are severely affected (Kison and Seemüller, 2001; Torres et al. 2004), whereas *Prunus domestica, Prunus cerasifera* and *Prunus insititia* are found to be less affected (Kison and Seemüller 2001). Differences in response to ESFY infections also occur between cultivars within species (Koncz et al. 2017; Marcone et al. 1996; Richter 2002). Diverse symptoms are known to be associated with phytoplasma diseases. Besides structural changes of the vascular system, such as callose deposition, phloem necrosis, and hyperplasia (Musetti et al. 2016; Zimmermann et al. 2015), phytoplasma infections affect translocation of carbohydrates between source and sink plant organs and alter the metabolic compositions of leaf tissue (Christensen et al. 2005; Lepka et al. 1999; Prezelj et al. 2016). Because phytoplasmas are obligate parasites depending on their host plants and insects, they have small genomes that lack genes for some metabolic pathways and need to obtain nutrients from the host organism (Bai et al. 2006; Kube et al. 2008; Marcone et al. 1996; Marcone et al. 1999). While several studies highlighted changes in the chemistry of plant tissue due to phytoplasma infections, only few studies have determined the effects on the chemical composition of the phloem, which is the side of infection. In the most recent publication comparing the phloem composition of phytoplasma-infected vs. non-infected mulberry plants, Gai et al. (2014) found a change in the metabolic composition of phloem sap in response to phytoplasma infection. Their analysis revealed higher amounts of sucrose, abscisic acid (ABA), cytokinin and total content of free amino acids in phloem sap from infected than non-infected plants. In contrast, the phloem metabolome of coconut palms was not affected by lethal yellowing disease (Stemmer et al. 1982).

‘*Ca*. P. prunorum’ and other phytoplasma species of the 16SrX or apple proliferation group are transmitted by jumping plant lice or psyllids of the superfamily Psylloidea (Hemiptera: Sternorrhyncha) feeding on plant sieve tube elements (Weintraub and Beanland 2006). These psyllid-transmitted phytoplasmas as well as their vectors are closely related and associated with economically important diseases of fruit trees such as pear decline, apple proliferation and ESFY (Jarausch et al. 2019). The plum psyllid *Cacopsylla pruni* transmits ‘*Ca*. P. prunorum’, the causal agent of ESFY by feeding on the phloem tissue of plants during reproduction (Carraro et al. 1998, 2004b). Little is known about the influence of phytoplasma infections on the nutritional composition of phloem and consequences on vector behavior and development. Amino acid composition, plant defense mechanisms and phytohormone concentrations (Dermastia 2019) could affect insect vector feeding on diseased plants. Although it is well known that nutritional quality and hormonal levels of plants in general impact insect performance and fitness (Cao et al. 2016; Pradit et al. 2019; Schoonhoven et al. 2010; Schweiger et al. 2014), much less is known about how plant infections with phloem-restricted bacteria impact insect fitness.

*Cacopsylla picta* emigrants that developed on *Malus domestica* trees infected with ‘*Ca*. P. mali’ are smaller and their development is slightly elongated compared to psyllids that develop on healthy apple plants (Mayer et al. 2011). Consequently, females prefer healthy over infected plants for oviposition (Mayer et al. 2011). In contrast, the survival and reproduction of female *Macrosteles quadrilineatus* and *Dalbulus maidis* is enhanced on host plants infected with Aster Yellows-witches’ broom phytoplasma (AY-WB) (Beanland et al. 2000; Purcell 1988; Sugio et al. 2011), while the infection of host plants with Bois Noir has no impact on growth of vector progeny (Kaul et al. 2009). By investigating the feeding behavior of Asian citrus psyllid (*Diaphorina citri*) with electropenetrography (EPG), Cen et al. (2012) recorded lower mean durations of phloem ingestion phase (E2) on plants inoculated with *‘Candidatus* Liberibacter asiaticus’ (CLas) than on uninfected plants. George et al. (2018) revealed lower total durations of E2 per psyllid in infected than uninfected *Citrus* plants. This reduction of phloem uptake is in accordance with the elongated developmental time of *D. citri* nymphs when reared on CLas-infected compared to uninfected *Citrus* plants (Pelz-Stelinski et al. 2010).

*Prunus persica* is highly susceptible to ESFY and shows severe symptoms and high mortality, while *P. insititia* is also susceptible but shows light symptoms and low mortality (Kison and Seemüller 2001). Therefore, we expected a significant influence of ‘*Ca*. P. prunorum’ on the phloem metabolome of *P. persica.* A comparison with the metabolite composition of infected *P. insititia* could indicate whether phloem chemistry is influencing symptom manifestation or reveal components associated with phytoplasma tolerance. Killiny and Hijaz (2016) found higher abundance of amino acids involved in plant defense mechanisms in phloem sap of citrus varieties tolerant to CLas.

To investigate the interaction of ‘*Ca*. P. prunorum’ with its natural plant environment, we analyzed sugars, sugar alcohols and organic acids in phloem centrifugates of infected and non-infected *Prunus* trees. Furthermore, we compared two *Prunus* species, which were differently affected by the infection (*P. persica* and *P. insititia*). To link the composition of primary plant metabolites of phloem centrifugates with vector development, we recorded and analyzed the feeding behavior and development of *C. pruni* nymphs on healthy and ‘*Ca*. P. prunorum’-infected plants. The importance of volatile organic compounds released by plants on *C. pruni* host preference and the importance of phloem chemistry on *C. pruni* development has been addressed previously (Gallinger et al. 2019, Gallinger and Gross 2018). Thus, the objective of the present research was to investigate the importance of gustatory cues on the host plant choice of *C. pruni* using two *Prunus* species that exhibit different degrees of sensitivity to ESFY phytoplasma infection.

## Methods and Materials

### Insects

Overwintered *C. pruni* adults (remigrants) were collected by beating foliage above a collection tray in early spring (March and April). Psyllids were sampled at two different sites: the experimental field and surroundings of the Julius Kühn-Institut (JKI) in Dossenheim, Germany, and an experimental *Prunus* orchard of Dienstleistungszentrum Ländlicher Raum Rheinpfalz (DLR), Neustadt an der Weinstrasse, Germany. Psyllids were reared on *Prunus spinosa* trees in insect cages (BugDorm, MegaView Science Co, Taiwan 47.5 × 47.5 × 93 cm), housed in a climate chamber at 20 °C (photophase) and 16 °C (scotophase) (L16:D8).

### Plants

Cultivars of *P. persica* (cv. South Haven) and *P. insititia* (cv. GF655–2) were used for experiments. *P. insititia* (cv. GF655–2) plants were dug out in October from the experimental field of the JKI and used for the experiments. Scion wood of *P. persica* cv. South Haven was grafted on one-year-old peach seedlings (cv. Montclar) as is common practice in fruit growing. All plants were grown in 1.8 L pots with clay substrate (Klasmann-Dielmann GmbH, Geeste, Germany). Plants were fertilized with ~ 500 ml Triabon (Compo Expert GmbH, Münster, Germany, 2 g/L) once in March and then weekly with 300–500 mL Wuxal (Hauert MANNA Düngerwerke GmbH, Nürnberg, Germany, 0.2%). *Prunus* trees were treated once with paraffin oil in March to prevent infestations with spider mites. All plants were housed in an insect free environment and treated weekly with nematodes *Steinernema feltiae* (SAUTTER & STEPPER GmbH, Ammerbuch, Germany) against fungus gnats. Polymerase chain reaction (PCR) analysis revealed naturally occurring phytoplasma infections in *P. insititia* plants from the field. Because we had no naturally infected *P. persica* plants, *P. persica* trees were graft-inoculated with *‘Ca*. P. prunorum’ ESFY Q06 from *Prunus marianna* GF 8–1 (*Prunus cerasifera x Prunus munsoniana*). Each tree was inoculated with two side-graftings of infected scion wood. Phytoplasma infestation was verified via PCR prior to experiments. Plants that were inoculated but infection with ‘*Ca*. P. prunorum’ could not be verified were excluded from the experiments. Experiments were conducted between May and August in 2018 and 2019 during leaf and shoot development. No plants expressed inflorescences during the two years of experiments.

### Development of C. pruni

The influence of ESFY infection and host species on developmental time of *C. pruni* was investigated. Therefore, nymphs were placed on healthy and ESFY-infected *P. persica* cv. South Haven and *P. insititia* cv. GF 655–2 plants. Second instar nymphs were gently transferred with a fine brush from a *P. spinosa* plant to middle-aged fully expanded leaves from experimental plants. Ten nymphs were placed on each leaf and were caged with small gauze bags (10 × 12 cm). Due to logistic reasons, seven to ten bags (70–100 nymphs) were attached to plants from each species and ESFY infection status. Bags were monitored daily for nymph development and adult eclosion. Eclosed adults were counted daily and removed from the bags. The experiment continued for 49 days until all adults eclosed or nymphs died. The experiment was set up in May and ended in July 2019. Plants were inoculated with phytoplasmas two years before the experiment.

### Electropenetrography (EPG)

Fifth instar nymphs were collected from the rearing cages with *P. spinosa* plants one hour before EPG recordings (1 h starvation period). Nymphs were carefully cleaned with a wet cotton stick and were allowed to dry for about 10 min. A droplet of water-based silver glue (EPG-Systems, Wageningen, The Netherlands) was attached to the mesothorax of each nymph and a piece of fine gold wire (18 μm diameter, ca. 1 cm length) was fixed on the pronotum with a second droplet of silver glue. The gold wire was connected to a copper extension wire soldered to a brass insect pin. The pin was attached to the EPG probe. The reference electrodes were placed into the wet soil of the test plants. The feeding behavior of *C. pruni* nymphs was recorded with an 8-channel amplifier (model Giga-8d, EPG-Systems, Wageningen, The Netherlands) in a climate chamber at 23 °C with 60%–65% RH for 16 h (log-day period). Nymphs were placed on the adaxial surface of mature leaves (second to sixth fully expanded leaves). Plants and insects were housed in a grounded self-constructed Faraday cage made of zinc-coated bird cage wire (mesh size: 6.3 × 6.3 mm) during the recordings. Feeding patterns of 15 individuals were recorded from both ESFY-infected and non-infected *P. insititia* and *P. persica* plants. Only recordings from nymphs that showed 16 h of activity were included in the analysis, while nymphs that molted during the experiment were excluded. EPGs were recorded in May and June one and two years after inoculation with phytoplasmas (2018 and 2019). Data acquisition and analysis was performed with Stylet+ software (EPG-Systems, Wageningen, The Netherlands). Recordings were examined for occurrence of waveforms according to Bonani et al. (2010) and Civolani et al. (2011). Patterns corresponding to the start of penetration and the stylet position in the parenchyma (A, B, C1 and C2) were summarized as intracellular pathway phase (C). The phase between the parenchyma and the phloem was considered at phase D, which has been suggested as the transition phase between parenchyma and phloem. The two phloem feeding waveforms were E1 and E2, while the ingestion of xylem content was G. Finally, the non-probing (Np) phases were also annotated during which time insects were not penetrating the plant tissue with their stylets.

### Collection of Sap Samples

One phloem sap sample was collected from each tree with the centrifugation technique according to Hijaz and Killiny (2014). Briefly, the bark from young flush of *P. persica* and *P. insititia* plants was removed manually and sliced into 1–2 cm pieces with a clean scalpel. The bottom of a 0.5 ml Eppendorf tube was removed. Each tube was immersed in a second, larger tube (1.5 ml). To collect the phloem content, bark pieces were placed into the small tube and centrifuged at 12.000 rpm at 4 °C for 10 min. The collected samples were stored at −80 °C until analysis. As we cannot totally exclude possible slight contamination from mesophyll cell content, we refer to the samples as phloem centrifugates henceforth. Phloem centrifugates were sampled in August 2018 one year after inoculation with phytoplasmas.

### Measurement of °Brix Value

To compare the absolute amount of soluble solid content in phloem centrifugates, °Brix values were measured with a handheld refractometer (type 45–81; Bellingham + Stanley Ltd., Tunbridge Wells, UK). The refractometer was calibrated with sucrose as standard. About 1 μl phloem centrifugate from either *P. insititia* (n_non-infected_ = 6_,_ n_infected_ = 6) or *P. persica* (n_non-infected_ = 11, n_infected_ = 7) were used for measurements.

### Derivatization of Phloem Centrifugates

Silylation was used to analyze sugars, sugar derivates and organic acids in phloem centrifugates. Five μl of the samples were added to 60 μl of a 1.5 mmol ribitol internal standard solution (Sigma-Aldrich Chemie GmbH, Munich, Germany) and dried under nitrogen stream (Reacti-Vap, Thermo Fisher Scientific Inc., Waltham, Massachusetts, USA). Seventy μl of methoxyamine hydrochloride solution (MOX) in pyridine (2%) was added to each sample. Methoxyamine was allowed to react for 90 min at 37 °C stirring at adjustment of 7 (Reacti-Therm, Thermo Fisher Scientific Inc.). N-methyl—(N-trimethylsilyl) (MSTFA) was used as silylation reagent. After adding 90 μl MSTFA to each sample, the reaction was incubated for 60 min at 37 °C with stirring at adjustment of 7. The supernatant was transferred to a GC-MS vial with a glass insert. A second derivatization method using methyl chloroformate was used to optimize the detection of amino acids (Smart et al. 2010). Aliquots of 15 μl phloem centrifugates were mixed with 7.5 μl DL-norvaline (Sigma-Aldrich Chemie GmbH) as an internal standard (17 mmol in ultrapure water) and 180 μl sodium hydroxide (1 M). 167 μl methanol and 34 μl pyridine were added, followed by 20 μl MCF. Afterwards, the sample was vortexed for 30 s., and an additional 20 μl of MCF were added and the sample was mixed for 30 s. again. The alkylated derivatives were extracted by adding 150 μl chloroform and mixing for 10 s. After adding a 200 μl aliquot of sodium bicarbonate solution (50 mM), the samples were mixed again for 10 s. Silanized glass vials (Carl Roth GmbH + Co. KG, Karlsruhe, Germany) were used for the chemical reaction. The aqueous phase was discarded. To bind any remaining water, a few milligrams of anhydrous sodium sulfate were added to the organic layer. The supernatant was transferred to a GC-MS vial with a glass insert.

### GC-MS Analysis

Derivatized samples were analyzed by gas chromatography coupled with mass spectrometry (GC-MS) using a PerkinElmer Clarus R 680 GC system coupled to a Perkin Elmer quadrupole inert mass selective detector. For GC separation a nonpolar Elite-5MS (Crossbond 5% diphenyl −95% dimethyl polysiloxane, PerkinElmer) capillary column (30 × 0.25 mm id × 0.25 μm film thickness) was used. One μl of samples derivatized with MCF were injected with an open injector vent at 70 °C injector temperature, to purge out the solvent. After 0.5 min, the vent was closed and the injector temperature was raised to 290 °C after 1 min. Carrier gas flow rate (Helium, Air Liquide, Germany) was about 5 ml/min (column head pressure 130 kPa) and 30 ml/min split flow. The initial oven temperature of 80 °C was held for 2 min, followed by a temperature increase of 10 K/min up to 240 °C held for 3.5 min and a further increase to 300 °C at a rate of 20 K/min. The final temperature of 300 °C was held for 2 min. For the analysis of sap samples after silylation, 1.5 μl of each sample was injected with a split flow of 5 ml/min at 140 °C and the injector temperature was increased by 50 K/min to 250 °C. Column head pressure of Helium flow was set to 130 kPa. The GC temperature program was as follows: the initial oven temperature of 80 °C was held for 3 min, followed by an increase of 5 K/min up to 320 °C. The final temperature of 320 °C was held for 4 min. For all analysis the transfer line and ion source temperatures were set to 250 °C and 180 °C respectively. The quadrupole mass detector was operated in electron-impact (EI) mode at 70 eV. All data was obtained by collecting the full-scan mass spectra within the range of 35–550 m/z. Blank samples, reference standards and mixtures of alkanes (C8 - C20 and C 10- C40) were analyzed additionally according to both methods. Reference standards and suppliers are listed in the supplementary material (Table S1).

### Identification and Quantification with AMDIS

Chromatograms of sap sample derivates were analyzed using “Automated Mass spectral Deconvolution and Identification System” (AMDIS, V. 2.71; National Institute of Standards and Technology NIST, Boulder, CO). For the identification, the ion fragmentation patterns and retention indices of detected compounds were compared with standard compounds (Gross et al. 2019). Compounds that were not identified were annotated as unknowns. For quantification, the peak areas were integrated after deconvolution. Identification criteria were applied as follows: match factor had to be ≥ 80% and the relative retention index deviation ≤ 5% from reference value. The settings for deconvolution were: component width: 32; adjacent peak subtraction: one; resolution: medium; sensitivity: medium; shape requirements: low; level: very strong; maximum penalty: 20 and ‘no RI in library’: 20. Components with a signal to noise ratio < 50 were excluded from the analysis. Relative amounts of detected compounds after derivatization were calculated in relation to the respective internal standards norvaline and ribitol.

### Statistical Analyses

All statistical analyses were conducted in R version 3.5.3 (R Core Team 2017). Graphics were produced using the ggplot2 package (Wickham 2009). A parametric survival model (time-to-event analysis) was used to investigate the effect of plant species and the infection status of plants on the development of *C. pruni*. The model was fitted with an exponential distribution with the *survreg* function of the ‘survival’ package. Linear models (LMs) were used to determine the influence of the plant species and phytoplasma infection on the duration of waveforms per event (total), duration per nymph (mean) and the time to first occurrence of waveforms in EPG recordings from *C. pruni* nymphs. In case of non-normality of residuals, the data were transformed as specified in Table S2. The fit of models with the main effects ‘*Prunus* species’ and ‘ESFY infection status’ and the interaction of these two factors was compared by second-order Akaike’s information criterion (AICc) corrected for small samples. To analyze the occurrence (frequency) of individual waveforms per nymph, GLMs with quasi-Poisson distribution were used due to overdispersion. To compare models fitted with quasi-Poisson distribution the quasi-AICc (qAICc) was computed, using the model deviance instead of the likelihood and used in the *ICtab* function from ‘bbmle’ package (Bolker and R Development Core Team 2017). A LM was fitted with square root transformed °brix values, to analyze the influence of *Prunus* species and ESFY infection on the amount of total soluble solid content in phloem centrifugates. AICc was used to identify best model fit. Model assumptions were validated graphically as recommended by Zuur et al. (2009). The *emmeans* function from the ‘emmeans’ package (Lenth et al. 2019) was used to calculate the estimated marginal means and corresponding 95% confidence intervals and to determine differences between treatment levels. In case multiple pairwise comparison *p*-values were adjusted by the method of Tukey. Discrimination of the chemical composition of phloem centrifugates from infected and non-infected *Prunus* trees was calculated by a type II permutation multivariate analysis of variance (PERMANOVA) of the Bray-Curtis dissimilarities matrix. The PERMANOVA was calculated with the *adonis.II* function from ‘RVAideMemoire’ package (Hervé 2019). The dispersions of groups were tested for multivariate homogeneity (PERMDISP). Both analyses were calculated with *N* = 10000 permutations. The Bray–Curtis dissimilarities were visualized by non-metric multidimensional scaling (NMDS) plots. The scaling was standardized by Wisconsin double standardization and performed using the *metaMDS* function from ‘vegan’ package (Oksanen et al. 2019). Influence of main factors and interaction on the relative amount of total amino acids, sugars, sugar alcohols, and organic acids were analyzed by fitting linear models as described above. The model specifications were as reported in Table S3.

## Results

### Development of C. pruni

**Fig. 1:**
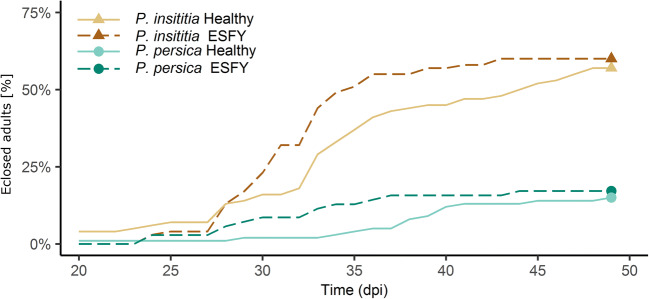
Cumulative percentage of *C. pruni* nymphs completing development per day post infestation (dpi) on ESFY infected and healthy *P. insititia* (n_healthy_ = 100, n_ESFY_ = 100) and *P. persica* (n_healthy_ = 100, n_ESFY_ = 70) trees. Ten nymphs were caged together on each leaf

After 49 days, all *C. pruni* nymphs had emerged to adults or died (Fig. 1). The development of *C. pruni* was significantly different between both *Prunus* species (*survreg*, *Z* = 7.09*, df* = 1, *P* < 0.01 *N* = 370). Fifty-seven and 60% of nymphs developed on healthy and phytoplasma-infected *P. insititia* plants, respectively; whereas, 15% of *C. pruni* emigrants emerged on healthy and 12% on diseased *P. persica* trees. Mean development time was 41 and 39 days on healthy and infected *P. insititia* plants, respectively. On average, *C. pruni* nymphs required 47 days for development on *P. persica* plants. Phytoplasma infection had no significant influence on the development of *C. pruni* nymphs (*survreg*, *Z* = 0.34*, df* = 1, *P* = 0.73, *N* = 370).

### EPG

Waveforms detected in EPG recordings from *C. pruni* nymphs were comparable to those specified for *C. pyri* (Civolani et al. 2011). The intracellular pathway phase (C), a phase that always occurred between parenchyma and phloem phases (D), two phloem patterns (E1 and E2), a xylem pattern (G) and non-probing phases (Np), as described by Civolani et al. (2011), were identified in the recordings.

### Frequency

The mean number of waveforms C, E2 and Np phases were neither affected by the *Prunus* species nor by infection status of the plants. Whereas the main effect of the plant species was significant for the occurrence of waveform D (*GLM*, *χ*^*2*^ = 9.56, *df* = 1, *P* = 0.002, *N* = 60) and E1 (*GLM*, *χ*^*2*^ = 4.96, *df* = 1, *P* = 0.026, N = 60), both waveforms were recorded more frequently from nymphs feeding on *P. persica* than on *P. insititia* plants (Table 1). The number of bouts of G was influenced by the infection status of the plants (*GLM*, *χ*^*2*^ = 4.03, *df* = 1, *P* = 0.044, *N* = 60). On average, nymphs accessed the xylem of healthy leaves 4 ± 3.27 and the xylem of infected leaves 2.7 ± 1.73 times during the 16 h recording period on both *Prunus* species (Table 1).

**Table 1:** Frequency of waveform events occurring in 16 h EPG recordings of *C. pruni* nymphs on *P. insititia* (n_healthy_ = 15, n_ESFY_ = 15) and *P. persica* (n_healthy_ = 15, n_ESFY_ = 15) trees

Waveform		*P. insititia*		*P. persica*		*P. insititia*	*P. persica*	model statistics*		
Frequency		mean ± SE	(min-max)	mean ± SE	(min-max)	emmean (lower-upper CI)	emmean (lower-upper CI)	influential factors	*χ*^*2*^	P
C	healthy	35.87 ± 5.3	(5–73)	34.87 ± 3.06	(20–57)					
	ESFY	33.07 ± 4.69	(8–65)	39.27 ± 3.49	(20–58)					
D	healthy	7 ± 1.75	(1–29)	12.2 ± 1.27	(3–18)	6.77 (5.11–8.97)	11.70 (9.45–14.49)	species	9.560	0.002
	ESFY	6.53 ± 1.06	(1–14)	11.2 ± 2.15	(0–29)					
E1	healthy	11.07 ± 2.87	(1–48)	16.33 ± 1.82	(3–27)	10.8 (8.16–14.4)	16.3 (12.97–20.6	species	4.959	0.026
	ESFY	10.6 ± 1.8	(1–23)	16.33 ± 3.16	(0–34)					
E2	healthy	6.33 ± 1.8	(1–30)	8.07 ± 1.1	(1–16)					
	ESFY	6.53 ± 1.23	(1–17)	9.2 ± 2.2	(0–28)					
G	healthy	3.47 ± 0.75	(1–13)	4.53 ± 0.93	(1–15)	4.0 (3.13–5.11)				
	ESFY	2.2 ± 0.45	(0–6)	3.2 ± 0.42	(1–6)	2.7 (2.00–3.64)		infection	4.035	0.044
np	healthy	24.73 ± 3.66	(3–54)	17.47 ± 1.94	(4–32)					
	ESFY	23.07 ± 4.52	(3–62)	24.33 ± 3.11	(9–50)					

Mean (± SE) number per nymph, value range of occurrence and significant effects of *Prunus* species, ESFY infection of *Prunus* trees on the number of events. The estimated marginal means and the corresponding confidence intervals from the models are shown for significant factors

* Generalized linear models with quasi-Poisson distribution were used to analyze the effects of main factors and interactions on the frequency of waveforms events. Model statistics are presented for models simplified by removing nonsignificant factors due to AICc.

### Mean Duration Per Psyllid

**Fig. 2:**
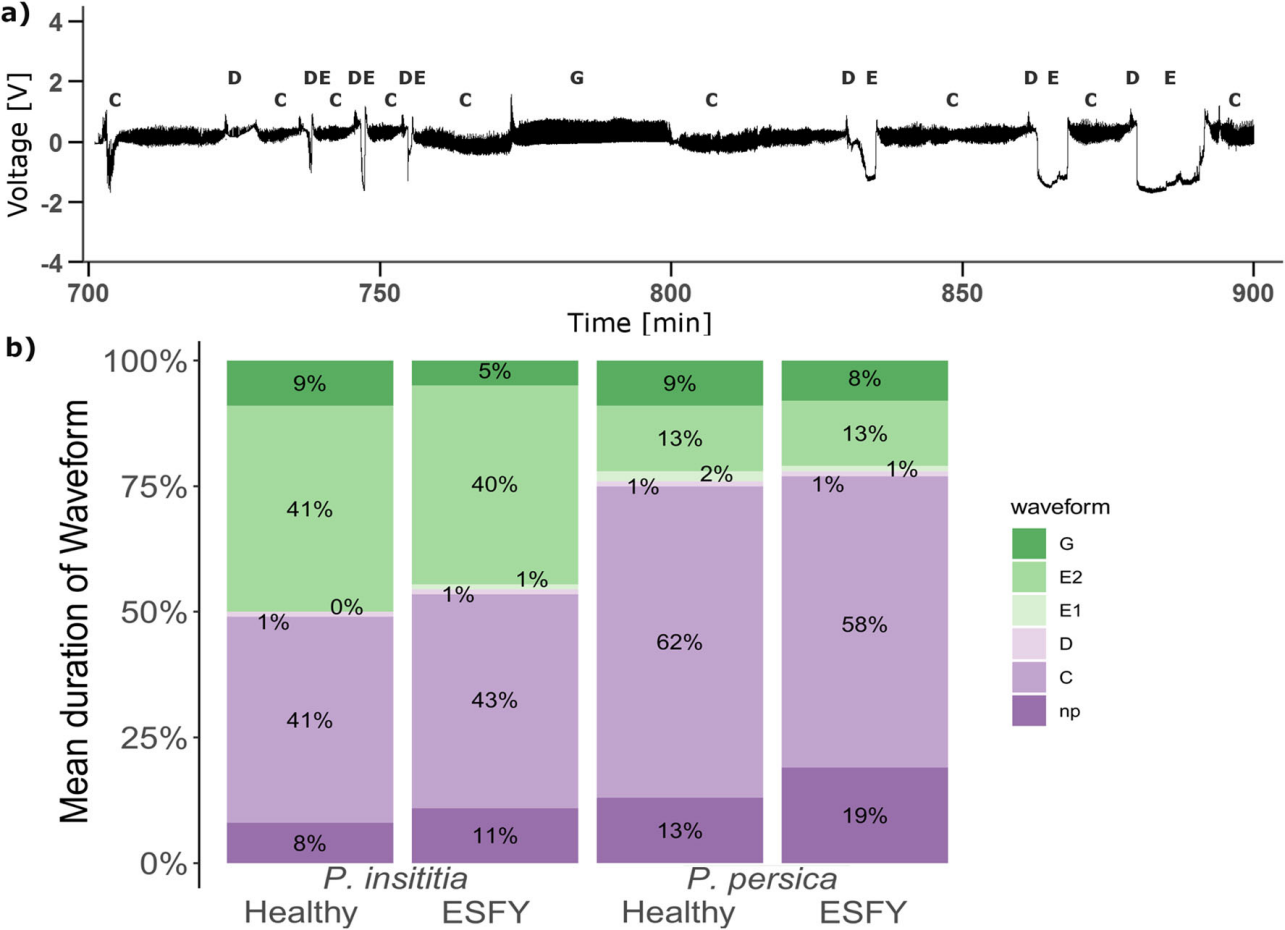
a) Example of electropenetrography recording from *C. pruni* nymphs on a non-infected *P. persica* plant showing the classified waveforms: Intracellular pathway phase (C), transition phase between the parenchyma and the phloem (D), phloem salvation and ingestion (E), ingestion of xylem content (G) and the non-probing (Np) phases. b) Mean percentage duration of waveforms per psyllid detected during 16 h EPG recordings with *C. pruni* nymphs on *P. insititia* (n_healthy_ = 15, n_ESFY_ = 15) and *P. persica* (n_healthy_ = 15, n_ESFY_ = 15) trees. Additional explanations to particular waveforms are given in the text

Plant species had a strong effect on the mean duration of C (*GLM*, *F* = 12.38, *df* = 1, *P* = <0.001, *N* = 60), D (*GLM*, *F* = 11.32, *df* = 1, *P* = 0.001, N = 60), E1 (*GLM*, *F* = 12.27, *df* = 1, *P* = <0.001, N = 60), E2 (*GLM*, *F* = 21.80, *df* = 1, *P* = <0.001, N = 60) and Np (*GLM*, *F* = 5.75, *df* = 1, *P* = <0.02, N = 60) phases (Table 2). The mean duration per nymph in the pathway phase and phases (C) of non-probing (Np) were significantly longer during feeding on *P. persica* than on *P. insititia* (Table 2). Furthermore, the durations of D and E1 were longer on *P. persica* than *P. insititia* plants (Table 2). Nymphs feeding on *P. persica* plants spent about 75% of the time in non-ingestion phases, 13% ingesting phloem and 9% ingesting xylem. In contrast, nymphs feeding on *P. insititia* ingested phloem three times longer and the time spent in non-feeding phases was 50% lower than on *P. persica* (Fig. 2b).

**Table 2:** Waveform durations per nymph from 16 h EPG recordings of *C. pruni* nymphs on *P. insititia* (n_healthy_ = 15, n_ESFY_ = 15) and *P. persica* (n_healthy_ = 15, n_ESFY_ = 15) trees

Waveform		*P. insititia*		*P. persica*		*P. insititia*	*P. persica*	model statistics*		
Duration / Nymph [min]		mean ± SE	(min-max)	mean ± SE	(min-max)	emmean (lower-upper CI)	emmean (lower-upper CI)	influencial factors	F	P
C	healthy	388.73 ± 56.44	(53.21–745.35)	584.07 ± 50.81	(200–858.99)	371 (309–439)	550 (474–632)	species	12.376	< .001
	ESFY	409.62 ± 47.86	(166.03–771.73)	542.98 ± 34.54	(317.31–767.75)					
D	healthy	6.33 ± 1.27	(1.36–18.6)	14.03 ± 1.79	(3.77–33.53)	5.49 (3.69–7.65)	11.21 (8.55–14.21)	species	11.321	0.001
	ESFY	6.11 ± 1.19	(1.27–19.1)	12.09 ± 2.99	(0–38.45)					
E1	healthy	4.17 ± 0.83	(0.91–11.85)	17.05 ± 4.56	(1.48–64.91)	4.77 (3.53–6.45)	10.04 (7.44–13.57)	species	12.27	< .001
	ESFY	5.53 ± 1.18	(0.22–17.26)	10.95 ± 2.29	(0–28.97)					
E2	healthy	386.22 ± 73.84	(3.79–798.54)	125.54 ± 38.62	(0.93–469.65)	382 (303.4–460)	124 (45.9–202)	species	21.798	< .001
	ESFY	376.83 ± 64.52	(12.17–741.01)	122.38 ± 38.7	(0–520.23)					
G	healthy	87.36 ± 28.1	(19.66–460.72)	88.89 ± 17.6	(21.02–233.72)	66.1 (46.9–93.0)				
	ESFY	50.71 ± 12.67	(0–172.48)	72.05 ± 16.83	(6.68–273.92)	40.2 (28.6–56.7)		infection	4.203	0.045
np	healthy	80.34 ± 12.97	(17.29–202.36)	119.34 ± 20.68	(39.84–290.29)	80.6 (56.8–108)	130.3 (99.5–165)	species	5.754	0.020
	ESFY	102.04 ± 20.75	(3.62–331.89)	180.12 ± 35.29	(35.67–496.26)					

Mean (± SE) duration, value range of occurrence and significant effects of *Prunus* species, ESFY infection of *Prunus* trees on the duration per nymph. The estimated marginal means and the corresponding confidence intervals from the models are shown for significant factors

*Linear models were used to analyze the effects of main factors and interactions on the frequency of waveforms events. Model statistics are presented for models simplified by removing nonsignificant factors due to AICc

### Mean Duration Per Event

**Fig. 3:**
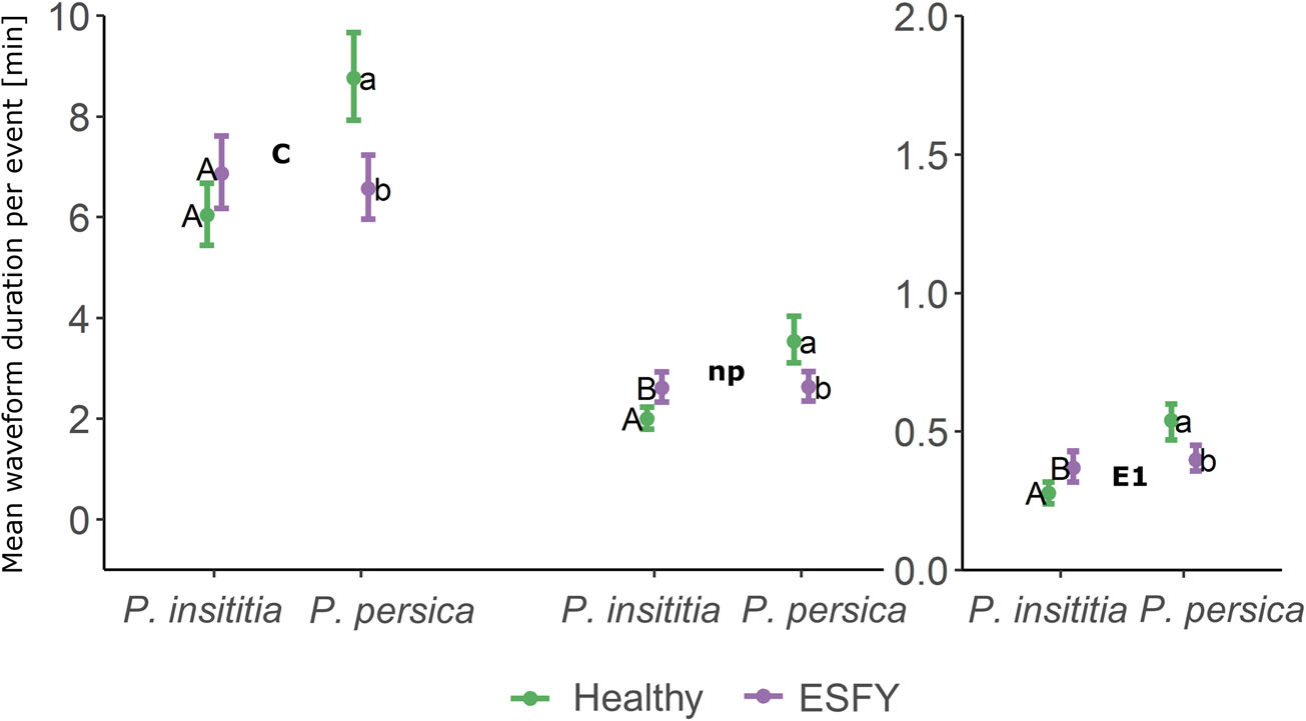
Interaction plots of estimated marginal means and confidence intervals predicted from linear models of the mean duration per event of the waveform C, Np and E1 from EPG recordings of *C. pruni* nymphs feeding on healthy or ESFY infected *P. insititia* (n_healthy_ = 15, n_ESFY_ = 15) and *P. persica* (n_healthy_ = 15, n_ESFY_ = 15) trees

*Prunus* species and the interaction between species and ESFY infection status significantly affected the mean duration of waveforms not associated with phloem ingestion (C, D, E1 and Np) (Fig.3). C phases were shorter on infected than non-infected *P. persica* trees, whereas infection had no influence on the duration of C on *P. insititia* plants (Fig. 3). While the duration of E1 and Np phases was shorter on infected than non-infected *P. persica* plants, E1 and Np lasted longer in infected than in healthy *P. insititia* trees (Fig. 3). Phloem ingestion phases (E2) by nymphs feeding on *P. insititia* were significantly longer than those by nymphs feeding on *P. persica* (Table 3). On average, xylem phases lasted for 22.31 (± 2.13 SE) min. The mean duration per event was not affected by *Prunus* species nor ESFY infection (Table 3)

.

**Table 3:** Duration of waveform per event from 16 h EPG recordings of *C. pruni* nymphs on *P. insititia* (n_healthy_ = 15, n_ESFY_ = 15) and *P. persica* (n_healthy_ = 15, n_ESFY_ = 15) trees

Waveform		*P. insititia*		*P. persica*		*P. insititia*	*P. persica*	model statistics*		
Duration / Event [min]		mean ± SE	(min-max)	mean ± SE	(min-max)	emmean (lower-upper CI)	emmean (lower-upper CI)	influential factors	F	P
C	healthy	10.84 ± 0.67	(0.05–174.54)	16.75 ± 1.23	(0.04–266.82)	6.04 (5.44–6.68)	8.76 (7.93–9.67)	species	9.549	0.002
	ESFY	12.39 ± 0.76	(0.12–130.96)	13.83 ± 1.23	(0.04–516.39)	6.87 (6.18–7.62)	6.57 (5.96–7.24)	infection	2.958	0.085
								interaction	16.448	< .001
D	healthy	0.9 ± 0.06	(0.06–4)	1.15 ± 0.05	(0.38–5.56)	0.77 (0.70–0.85)	1.05 (0.97–1.13)	species	20.818	< .001
	ESFY	0.94 ± 0.04	(0.14–2.42)	1.08 ± 0.05	(0.05–5.23)	0.85 (0.77–0.94)	0.94 (0.87–1.02)	infection	0.487	0.486
								interaction	5.315	0.022
E1	healthy	0.38 ± 0.03	(0.02–2.22)	1.04 ± 0.1	(0.05–11.89)	0.28 (0.24–0.32)	0.54 (0.47–0.60)	species	28.861	< .001
	ESFY	0.52 ± 0.05	(0.03–4.41)	0.67 ± 0.06	(0.03–7.97)	0.37 (0.32–0.43)	0.40 (0.36–0.45)	infection	0.814	0.367
								interaction	16.199	< .001
E2	healthy	60.98 ± 15.21	(0.23–768.65)	15.56 ± 4.95	(0.21–461.88)	9.17 (6.93–12.12)	3.28 (2.54–4.25)	species	42.507	< .001
	ESFY	57.68 ± 11.99	(0.09–706.66)	13.3 ± 2.72	(0.15–300.18)	12.53 (9.50–16.52)	4.49 (3.51–5.74)	infection	4.075	0.044
G	healthy	25.2 ± 6.06	(0.15–272.73)	19.61 ± 2.66	(0.17–142.97)					
	ESFY	23.05 ± 4.47	(0.71–135.65)	22.52 ± 3.68	(0.2–146.13)					
np	healthy	3.25 ± 0.35	(0.05–100.44)	6.83 ± 0.91	(0.1–201.55)	2.00 (1.79–2.23)	3.54 (3.11–4.04)	species	20.775	< .001
	ESFY	4.42 ± 0.33	(0.08–58.74)	7.4 ± 1.27	(0.04–282.72)	2.61 (2.33–2.93)	2.63 (2.35–2.94)	infection	0.017	0.8963
								interaction	22.568	< .001

Mean (± SE) duration, value range and significant effects of *Prunus* species, ESFY infection of *Prunus* trees and their interaction on the duration per event. The estimated marginal means and the corresponding confidence intervals from the models are shown for significant factors

* Linear models were used to analyze the effects of main factors and interactions on the frequency of waveforms events. Model statistics are presented for models simplified by removing nonsignificant factors due to AICc

### Time to First Occurrence

The time until each waveform occurred the first time was not affected by plant species nor ESFY infection status of the plants (Table S4).

### Chemistry of Phloem Centrifugates

To estimate the total content of soluble sugar content, °Brix was measured from centrifugates. °Brix differed significantly as a function of *Prunus* species (*LM*, *F* = 6.32, *df* = 1; *P* = 0.019, *N* = 30). A higher °Brix value was measured in centrifugates from *P. insititia* plants (13.33 ± 1.76 SD) than from *P. persica* plants (11.33 ± 2.33 SD).

**Fig. 4:**
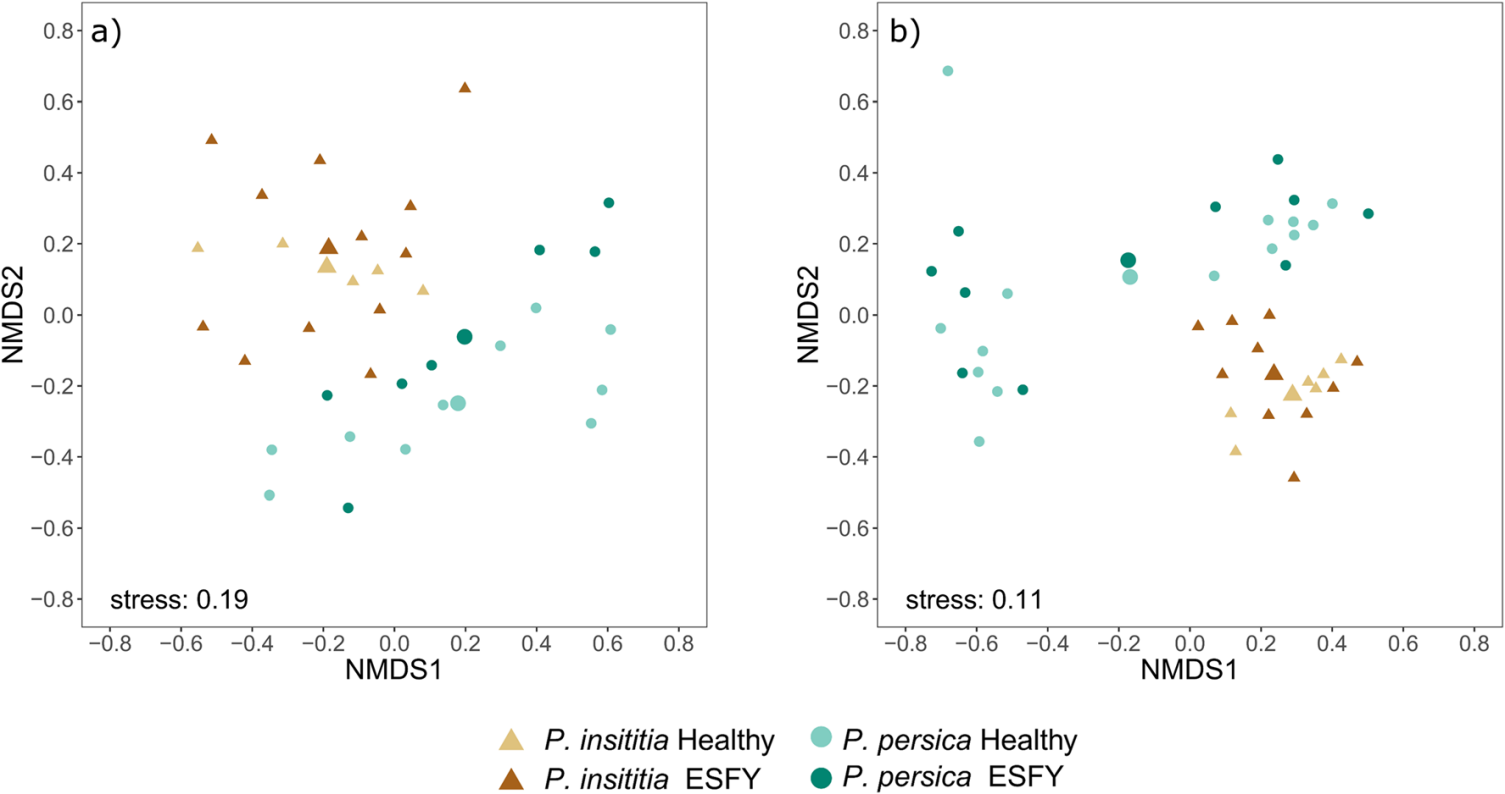
Visualization of Bray–Curtis dissimilarities with non-metric multidimensional scaling (NMDS) plots of phloem centrifugates from ESFY-infected (dark) and non-infected (light) *Prunus* trees. a) amino and other organic acids from *P. insititia* (brown triangles, n_healthy_ = 5, n_ESFY_ = 12) and *P. persica* (green dots, n_healthy_ = 10, n_ESFY_ = 7) trees and b) sugars and organic acids from *P. insititia* (n_healthy_ = 6 n_ESFY_ = 10) and *P. persica* (n_healthy_ = 14, n_ESFY_ = 10) trees. Large triangles and circles visualize group centroids

**Fig. 5:**
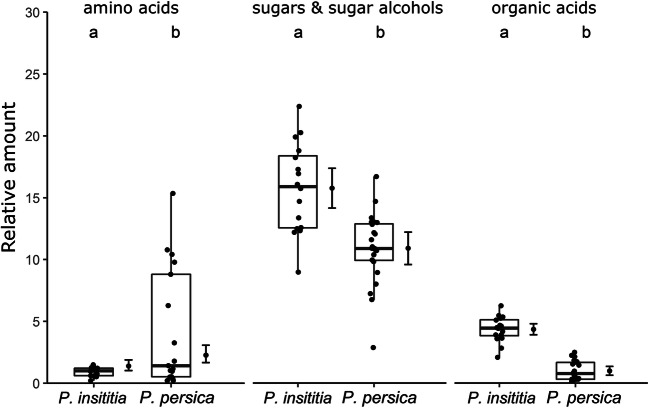
Mean relative amount of total amino acids of phloem centrifugates from *P. insititia* (*n* = 17) and *P. persica* (n = 17) after MCF derivatization, sugars and organic acids in phloem centrifugates from *P. insititia* (*n* = 16) and *P. persica* (*n* = 24) after silylation. Amino acids (asparagine, proline, aspartic acid, serine, glutamic acid, phenylalanine, lysine, histidine, tyrosine and tryptophan) have been quantified relative to the internal standard norvaline. Organic acids (phosphoric acid, malic acid, citric acid, quinic acid and galactaric acid), sugars and sugaralcohols (xylose, fructose, glucose, sucrose, mannitol, sorbitol and myo-inositol) after silylation have been quantified relative to internal standard ribitol. Boxes correspond to the 25th and 75th percentiles, medians are shown as lines, and whiskers extend to 1.5 times of the interquartile ranges. Dots represents raw values. Corresponding means and confidence intervals predicted for significant factors from linear models are shown on the right of each box

We found 10 amino acids and 9 organic acids (4 unidentified) in phloem centrifugates from *Prunus* trees after MCF derivatization (Table 4). The chemical composition of amino and organic acid in phloem centrifugates differed between the two *Prunus* species (Fig. 4a, *PERMANOVA*, *F* = 3.97, *df* = 1, *P* = 0.009, *N* = 34). The infection status as well as the interaction between infection and plant species had no significant effect on the discrimination between the phloem centrifugates (*PERMANOVA*, infection: *F* = 1.85, *d*f = 1, *P* = 0.117, interaction: *F* = 0.61, *df* = 1, *P* = 0.647, N = 34). The variance in samples from *P. persica* was significantly higher than from *P. insititia* (*PERMDISP*, *F* = 17.49, *df* = 1, *P* = 0.0002, N = 34). Higher relative amounts of caffeic acid and one unidentified compound (unknown_RI206) were detected in phloem centrifugates from *P. insititia* plants compared to *P. persica* plants (Table 4). High relative amounts of asparagine, glutamic acid, citric acid and one unknown compound (unknown_RI2062) were found in phloem centrifugates from *P. persica* trees (Table 4). Overall, phloem centrifugates from *P. persica* plants contained higher relative amounts of amino acids than those from *P. insititia* (Fig. 5).

**Table 4:**
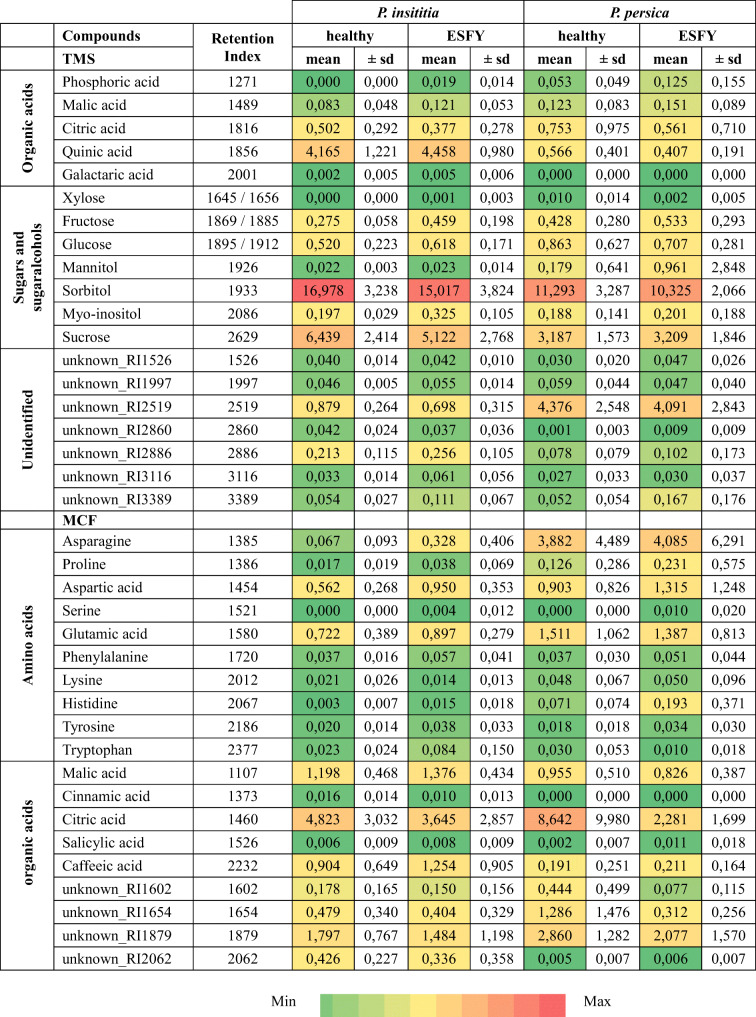
Mean relative amounts (± sd) of compounds detected via GC-MS analysis after derivatization

Amounts of organic acids, sugars, sugaralcohols and unknown compounds after silylation of phloem centrifugates from healthy or ESFY infected *P. insititia* (n_healthy_ = 6, n_ESFY_ = 10) and *P. persica* (n_healthy_ = 14, n_ESFY_ = 10) trees are relative to internal standard ribitol. Amounts of amino acids and organic acids after MCF derivatization of phloem centrifugates from healthy or ESFY infected *P. insititia* (n_healthy_ = 5, n_ESFY_ = 12) and *P. persica* (n_healthy_ = 10, n_ESFY_ = 7) trees are relative to the internal standard norvaline. Colors range from green (min) to red (max) (see below)

After TMS derivatization 5 organic acids, 7 sugars and sugar alcohols and 7 unidentified compounds were detected in phloem centrifugates (Table 4). The chemical composition of compounds after silylation differed significantly between the two *Prunus* species (Fig. 4b, *PERMANOVA*, *F* = 23.33, *df* = 1, *P* = 9e-05, *N* = 40); whereas, infection with ‘*Ca*. P. prunorum’ had no influence on the composition of the detected metabolites (*PERMANOVA*, *F* = 1.11, *df* = 1, *P* = 0.326, N = 40). The variability between all four groups did not differ (*PERMDISP*, *F* = 2.723, *df* = 3, *P* = 0.059, N = 40). In general, *P. persica* samples showed a greater variance than samples from *P. insititia* (*PERMDISP*, *F* = 4.891, *df* = 1, *P* = 0.033, N = 40). Sorbitol was the most abundant compound in phloem centrifugates from both *Prunus* species (Table 4). Phloem centrifugates from *P. insititia* contained more sorbitol, sucrose and quinic acid than those from *P. persica* plants (Table 4). However, larger quantities of unknown_RI2519 were detected in samples from *P. persica* than form *P. insititia* (Table 4)*.* The relative amount of sugars/sugar alcohols and organic acids was significantly higher in phloem centrifugates from *P. insititia* than from *P. insititia* plants (Fig. 5).

## Discussion

It was shown previously that the plum psyllid, *C. pruni*, prefers *P. insititia* plants over *P. persica* plants in field (Gallinger et al. 2019). Our current results suggest that avoidance of *P. persica* appears to be beneficial to *C. pruni*, given that nymphs feeding on *P. persica* exhibited prolonged developmental time and reduced developmental success than observed on *P. insititia*. In contrast, nymphs seem not to be repelled by *P. persica* plants because they initiated stylet penetration behavior as fast as that observed on *P. insititia*. This is in accordance with recent findings from olfactometer assays, showing that *C. pruni* exhibit no preference between *P. insititia* and *P. persica* plants based on olfactory cues (Gallinger et al. 2019). Waveform D, as recorded by EPG, is thought to reveal the transition from parenchyma to phloem tissue feeding (Civolani et al. 2011). Extended periods in D phase could be a result of structural characteristics of the vascular tissue, but *C. pruni* nymphs were able to reach the phloem of *P. persica* as often and as fast as that of *P. insititia*. Thus, mechanical barriers like sclerenchymatous rings surrounding the phloem, which are shown to inhibit adult *D. citri* from reaching the vascular tissue (Ammar et al. 2014), are unlikely to be involved in this system. Regardless, the duration of phloem-feeding by *C. pruni* was drastically reduced on *P. persica* compared to *P. insititia* plants. Therefore, we suggest that the feeding preference for *P. insititia* may be rather influenced by phloem chemistry than by mechanical barriers.

Analyses of phloem centrifugates revealed significant differences between the chemical composition of *P. persica* and *P. insititia.* We recorded higher brix values for phloem centrifugates of *P. insititia* than those for *P. persica*. GC-MS analysis revealed higher amounts of sucrose, sorbitol and quinic acid in phloem of *P. insititia* compared to *P. persica*. Although phloem is generally rich in nutrients, amino acids essential for insects are rare and phloem-feeders have to face the challenge of overabundance of carbohydrates and high osmotic pressures comprising their diets (Douglas 2006; Douglas et al. 2006).

In contrast to differences in feeding behavior pattern between the two plant species, phytoplasma infections solely significantly decreased in both *Prunus* species the duration of xylem ingestion. The same effect of bacterial infection (CLas) of *Citrus* plants on feeding behavior of *D. citri* was found using EPG studies (Cen et al. 2012; George et al. 2018). Typically*,* psyllid nymphs exhibit reduced xylem ingestion and prolonged phloem ingestion compared to adults to meet their nutritional requirements (Civolani et al. 2011; George et al. 2018). Interestingly, in our study the reduction of xylem ingestion was not associated with prolonged phloem ingestion. It is assumed that xylem ingestion by phloem-feeders helps regulate fluid balance (Spiller et al. 1990). For example, potato aphids (*Macrosiphum euphorbiae*) use ingestion of xylem content to regulate their osmotic potential (Pompon et al. 2011). The higher amount of soluble carbohydrates in *P. insititia* did not lead to an increased ingestion of xylem content by *C. pruni* nymphs feeding on *P. insititia*. Nymphs spent more time feeding on phloem and their mortality was lower on *P. insititia* plants, which contained fewer amino acids and higher amounts of sugars, than on *P. persica* plants, which had more amino acids and fewer sugars. The observed feeding behavior indicates that *C. pruni* is well adapted to *P. insititia* as a diet. Congruently, Jakobs and Müller (2018) documented that a high abundance of amino acids in phloem does not increase the developmental success of aphids in general. Instead, individual aphid species are adapted to specialized diet compositions (Jakobs et al. 2019). Interestingly, we found no increase in total sugar concentration (Brix value) in infected compared to non-infected plants. Thus, our results suggest that increased xylem phases are independent from phloem conditions and therefore might be based on differences in xylem metabolites.

Sugars can act as feeding stimulants for insects. The best-known example is sucrose, which stimulates feeding of many phytophagous insects, including aphids (Arn and Cleere 1971; Chapman 2003; Mittler and Dadd 1963). The sugar alcohol sorbitol is a characteristic phloem metabolite of plants belonging to the Rosaceae and could therefore play a central role in host acceptance of psyllid species feeding on *Prunus* spp., *Malus* spp. or *Pyrus* spp. (Spiraeoideae: Rosaceae)*.* The chemosensory sensilla from the mouthparts of *C. pruni* have not been described, but phagostimulatory cells that respond to sorbitol are known to occur in caterpillars specialized on rosaceaes plant species (Chapman 2003). Although sugars stimulate feeding by herbivores, phloem-feeders must excrete surplus non-assimilated sugars as honeydew (Ammar et al. 2013; Douglas 2006; Le Goff et al. 2019). Thus, future analysis of honeydew from nymphs could reveal components essential for proper development of *C. pruni* (Le Goff et al. 2019).

Of the compounds detected in the phloem centrifugates (Table 4), caffeic acid is of particular interest. Its possible positive influence on the feeding behavior of *C. pruni* deserves further investigation, because this metabolite was also detected in phloem sap of *Prunus domestica* but not in conifers, which are no suitable hosts for feeding and development of *C. pruni’s* offspring (Gallinger and Gross 2018). Hydroxycinnamic acids are commonly known as constitutive plant defenses against herbivores (Rehman et al. 2012). For example, chlorogenic acid is related to thrips resistance in plants (Leiss et al. 2009; Leiss et al. 2013). To our knowledge, the influence of phenolics on feeding behavior of psyllids has not been studied. Among psyllid species, phagostimulants have only been investigated for *D. citri* and appear to result from degradation products of common citrus volatiles (George et al. 2016; Lapointe et al. 2016). Therefore, further experiments should investigate whether reduced feeding of *C. pruni* nymphs is based on feeding deterrents or the lack of important metabolites that stimulate feeding on *P. persica.*

In the current study, we investigated the impact of phytoplasma infection on the phloem chemistry of its host plant. For this purpose, we compared two different plant-phytoplasma combinations: a less susceptible *Prunus* species naturally infected by a *‘Ca*. P. prunorum’ strain, which induced no symptoms, and a highly susceptible *Prunus* species inoculated with a ‘*Ca*. P. prunorum’ isolate that induces characteristic symptoms in the susceptible species. Neither type of infection with either ‘*Ca*. P. prunorum’ strain caused major changes in the composition of detected sugars, sugar alcohols and organic acids in *P. persica* or *P. insititia* plants. Naturally infested *P. insititia* plants were possibly colonized by a different phytoplasma strain than graft-inoculated *P. persica* plants. The virulence of phytoplasmas mainly depends on the combination of scions and isolates, but is also influenced by the rootstock. Kison and Seemüller (2001) investigated the virulence of different *‘Ca*. P. prunorum’ strains in combination with different *Prunus* species. *P. persica* scions and rootstocks suffered from infections with all tested ESFY isolates but to varying degrees. In contrast, *P. insititia* rootstocks have been less susceptible to all tested ESFY isolates. Regarding the current results, we cannot exclude the possibility that other combinations of scions, rootstocks and phytoplasma strains could affect changes to phloem composition. The feeding behavior of *C. pruni* nymphs was partly influenced by phytoplasma colonizing host plants. The interaction of the main factors (*Prunus* species and ESFY infection) affected feeding behavior. This supports the hypothesis that ESFY infections differentially affect *P. persica* and *P. insititia* trees. Shortened intracellular pathway phases (C) could indicate that *C. pruni* nymphs were able to reach the sieve-tube elements faster on infected *P. persica* than on uninfected plants. This might be a consequence of structural changes in phloem tissue, as enlargement of whole midribs is a characteristic symptom of ESFY in *P. persica* plants (Marcone et al. 1996).

Indeed, investigations have reported that *C. pruni* can survive and reproduce on *P. persica* in general (Carraro et al. 2004a; Fialová et al. 2004). However, we are the first to show that *P. persica* (peach) is a less suitable host for plum psyllids, which is clearly demonstrated by the low number of nymphs that developed successfully on *P. persica* plants. This is in accordance with findings from field surveys of *C. pruni* feeding on different *Prunus* species (Carraro et al. 2002; Gallinger et al. 2019; Mergenthaler et al. 2017). The measurement of abundance of *C. pruni* was monitored in these field surveys under the same conditions as in current study: non-grafted *P. insititia* rootstocks were compared with grafted *P. persica* scions on other rootstocks as this is common agricultural practice in fruit growing. To our knowledge there are no studies describing the influence of grafting on phloem chemistry of *Prunus* species, but it has been shown that rootstock species influences plant growth and fruit quality (Melnyk 2017). The rootstock-scion interaction can also influence psyllid feeding behavior, as grafting on resistant interstocks reduced scion susceptibility to pear psylla, *Cacopsylla bidens* (Shaltiel-Harpaz et al. 2018).

Even though *P. persica* is not a preferred host plant of *C. pruni,* trees are highly susceptible to phytoplasma infections and suffer from severe symptoms. Manifestation of symptoms could be elicited by physical changes of the vascular system and secondary metabolites, as an infection with *Ca*. P. prunorum’ induces the release of phytohormones and the deposition of callose in *P. persica* plants (unpublished data). Phytohormones could affect the feeding behavior of vector nymphs on ESFY-infected *P. persica* trees. There is evidence that plant defense mechanisms mediated by phytohormones are induced in response to *‘Ca*. P. prunorum’ infestations in apricot trees, which may lead to recovery from and tolerance to ESFY (Osler et al. 2014; Osler et al. 2016). Microbial phytopathogens induce hormonal changes in plants both directly and indirectly and this has been demonstrated for bacteria, fungi and viruses (Dermastia 2019; Killiny 2017; Ma and Ma 2016; Mauck et al. 2016). In many pathosystems these modifications are proven to alter the behavior of vector insect either directly or indirectly via volatile organic compounds (Bak et al. 2019; Martini et al. 2017; Martini et al. 2018; Mayer et al. 2008a, 2008b; Rid et al. 2016). Further, the infection status of the vector itself influenced the behavior (Mayer et al. 2008b). In this regard, the feeding and oviposition preferences of adult *C. pruni*, as influenced by their infection status, should be investigated to evaluate the possible effect on the transmission and spread of bacteria. Even though psyllid nymphs are less mobile than winged adults, nymphs spend more time feeding on phloem tissue (E1 and E2) (Civolani et al. 2011; George et al. 2018). As a result, acquisition of bacteria is higher when adults emerge from nymphs that fed on infected plants than when uninfected adults feed on infected plants (George et al. 2018; Inoue et al. 2009; Pelz-Stelinski et al. 2010). Consequently, transmission efficiency is higher when bacteria are acquired during the nymph than adult stage (Pelz-Stelinski et al. 2010). Since we found no negative effect of host plant phytoplasma colonization on development of *C. pruni* nymphs, it is possible that emerged adults contained high titers of bacteria and were capable of efficient pathogen inoculation.

## Electronic supplementary material

ESM 1 (DOCX 14 kb)

ESM 2 (DOCX 22 kb)
