# Supplementary material for: Phloem Metabolites of Prunus Sp. Rather than Infection with Candidatus Phytoplasma Prunorum Influence Feeding Behavior of Cacopsylla pruni Nymphs
Source: J Chem Ecol. 2020 Jan 22;46(8):756–70. doi: 10.1007/s10886-020-01148-8 (PMC7429536; doi:10.1007/s10886-020-01148-8)
Supplement: Supplementary file 1 — (DOCX 14 kb) [file 10886_2020_1148_MOESM1_ESM.docx]

Table S1: Suppliers of reference standards used for GC-MS analysis.

| Standard | Supplier |
| --- | --- |
| Alanine | Sigma-Aldrich Chemie GmbH  (Munich, Germany) |
| Aspartic acid |  |
| Cysteine |  |
| Glutaminic acid |  |
| Histidine |  |
| Iso-leucine |  |
| Leucine |  |
| Lysine |  |
| Myo-isonitol |  |
| Pinitol |  |
| Proline |  |
| Ribitol |  |
| Salicylic acid |  |
| Threonine |  |
| Tryptophan |  |
| Valine |  |
| Xylose |  |
| Arginine | SERVA Electrophoresis GmbH  (Heidelberg, Germany) |
| Phenylalanine |  |
| Glycine | Carl Roth GmbH + Co. KG  (Karlsruhe, Germany) |
| Methionine |  |
| Serin |  |
| Malic acid |  |
| Succinic acid |  |
| Arabinose |  |
| Sucrose |  |
| Asparagine | Merck KGaA  (Darmstadt, Germany) |
| Mannitol |  |
| Glucose |  |
| Galactose |  |
| Sorbitol | AppliChem GmbH  (Darmstadt, Germany) |
| Glutamine |  |
| Citric acid | Acros Organics  (Thermo Fisher Scientific, Geel, Belgium) |
