# Supplementary material for: Phloem Metabolites of Prunus Sp. Rather than Infection with Candidatus Phytoplasma Prunorum Influence Feeding Behavior of Cacopsylla pruni Nymphs
Source: J Chem Ecol. 2020 Jan 22;46(8):756–70. doi: 10.1007/s10886-020-01148-8 (PMC7429536; doi:10.1007/s10886-020-01148-8)
Supplement: Supplementary file 2 — (DOCX 22 kb) [file 10886_2020_1148_MOESM2_ESM.docx]

Table S2: Specification of linear models analyzing waveform parameters form EPG recordings of *C. pruni* nymphs (L5) feeding on healthy and ESFY infected *P. insititia* und *P. persica* leaves.

| waveform  mean duration / nymph | Data transformation | waveform  mean duration / event | Data transformation |
| --- | --- | --- | --- |
| C | sqrt | C | log+1 |
| D | sqrt | D | log+0.01 |
| E1 | log+1 | E1 | log+0.01 |
| E2 | none | E2 | log+0.01 |
| G | log+1 | G | log+10 |
| np | sqrt | np | log+0.01 |

Table S3: Specification and results of linear models analyzing the relative amount of total amino acids, sugars and organic acids in phloem sap samples from healthy and ESFY infected *P. insititia* und *P. persica.*

| Relative amount of | Data transformation | Factor | F-value | Pr(>F) |
| --- | --- | --- | --- | --- |
| Amino acids | log+0.01 | species | 4.376 | 0.044 |
| Sugars and sugaralcohols | none | species | 22.536 | <0.001 |
| Organic acids | none | species | 140.29 | <0.001 |

Table S4: Specification and results of linear models analyzing the time (min) until first occurrence of each waveform in EPG recordings from *C. pruni* nymphs (L5) feeding on healthy and ESFY infected *P. insititia* und *P. persica* leaves.

| Time to first occurrence of waveform | Data transformation | Factor | F-value  Full model | Pr(>F)  Full model | Best model  AICc |
| --- | --- | --- | --- | --- | --- |
| C | log+0.01 | species | 0.166 | 0.685 | null model |
|  |  | infection | 0.064 | 0.801 |  |
|  |  | interaction | 0.899 | 0.347 |  |
| D | sqrt | species | 1.712 | 0.196 | null model |
|  |  | infection | 0.025 | 0.874 |  |
|  |  | interaction | 0.196 | 0.660 |  |
| E1 | sqrt | species | 1.714 | 0.196 | null model |
|  |  | infection | 0.024 | 0.878 |  |
|  |  | interaction | 0.198 | 0.658 |  |
| E2 | sqrt | species | 2.129 | 0.151 | null model |
|  |  | infection | 1.722 | 0.195 |  |
|  |  | interaction | 0.906 | 0.345 |  |
| G | log+1 | species | 0.028 | 0.869 | null model |
|  |  | infection | 0.164 | 0.687 |  |
|  |  | interaction | 0.106 | 0.746 |  |
| np | log+1 | species | 0.463 | 0.499 | null model |
|  |  | infection | 0.293 | 0.590 |  |
|  |  | interaction | 0.027 | 0.870 |  |

| **waveform** | **P. insititia** | | | | **P. persica** | | | |
| --- | --- | --- | --- | --- | --- | --- | --- | --- |
| Time to first occurrence of waveform | **healthy** | | **ESFY** | | **healthy** | | **ESFY** | |
|  | **mean ± SE** | **(min-max)** | **mean ± SE** | **(min-max)** | **mean ± SE** | **(min-max)** | **mean ± SE** | **(min-max)** |
| **C** | 6.86 ± 3.03 | (0.64 - 37.61) | 9.14 ± 5.13 | (1.32 - 80.41) | 11.08 ± 6.41 | (1.2 - 99.49) | 19.42 ± 15.43 | (0.64 - 235.09) |
| **D** | 262.8 ± 56.83 | (50.25 - 897.45) | 280.8 ± 60.64 | (37.35 - 910.98) | 200.46 ± 30.52 | (75.72 - 512.48) | 232.83 ± 68.13 | (0 - 823.61) |
| **E1** | 263.86 ± 56.93 | (50.78 - 899.55) | 282.13 ± 60.69 | (38.02 - 912.76) | 201.52 ± 30.52 | (77.08 - 513.64) | 233.63 ± 68.12 | (0 - 824.38) |
| **E2** | 309.67 ± 57.56 | (50.93 - 900.46) | 293.74 ± 61.3 | (38.1 - 912.98) | 273.85 ± 47.62 | (77.29 - 684.07) | 210.24 ± 65.36 | (0 - 824.83) |
| **G** | 113.43 ± 27.21 | (12.12 - 437.6) | 196.46 ± 60.62 | (0 - 930.62) | 103.31 ± 23.24 | (12.08 - 275.94) | 196.26 ± 74 | (10.55 - 953.32) |
| **np** | 29.93 ± 10.77 | (1.17 - 159.48) | 27.98 ± 9.36 | (3.99 - 135.78) | 28.44 ± 9.04 | (2.28 - 116.64) | 43.21 ± 18.57 | (2.62 - 287.6) |
